# Recent advances in nanomaterial-based biosensor for periodontitis detection

**DOI:** 10.1186/s13036-024-00423-6

**Published:** 2024-04-18

**Authors:** Mohammad Hosseini Hooshiar, Masoud Amiri Moghaddam, Mohammad Kiarashi, Athraa Y. Al-Hijazi, Abbas Fadel Hussein, Hareth A.Alrikabi, Sara Salari, Samar Esmaelian, Hassan Mesgari, Saman Yasamineh

**Affiliations:** 1 Department of Periodontology, Tehran University of Medical Sciences, Tehran, Iran; 2 Assistant Professor of Periodontics, Dental Research Center, Mashhad University of Medical Sciences, Mashhad, Iran; 3 College of Dentistry, Lorestan University of Medical Sciences, Khorramabad, Iran; 4 College of Dentistry, Al-Mustaqbal University, Babylon, 51001 Iraq; 5 Department of Dentistry, Al-Noor University College, Nineveh, Iraq; 6 Collage of Dentist, National University of Science and Technology, Dhi Qar, 64001 Iraq; 7 Doctor of Dental Surgery, Islamic Azad University of Medical Sciences, Esfahan, Iran; 8 Faculty of Dentistry, Islamic Azad University, Tehran Branch, Tehran, Iran; 9 Department, Faculty of Dentistry Oral and Maxillofacial Surgery, Islamic Azad University, Tehran Branch, Tehran, Iran; 10 Young Researchers and Elite Club, Tabriz Branch, Islamic Azad University, Tabriz, Iran

**Keywords:** Periodontitis, Nanoparticles, Nanobiosensors, Detection, Biomarkers

## Abstract

Periodontitis, a chronic inflammatory condition caused by bacteria, often causes gradual destruction of the components that support teeth, such as the alveolar bone, cementum, periodontal ligament, and gingiva. This ultimately results in teeth becoming loose and eventually falling out. Timely identification has a crucial role in preventing and controlling its progression. Clinical measures are used to diagnose periodontitis. However, now, there is a hunt for alternative diagnostic and monitoring methods due to the progress of technology. Various biomarkers have been assessed using multiple bodily fluids as sample sources. Furthermore, conventional periodontal categorization factors do not provide significant insights into the present disease activity, severity and amount of tissue damage, future development, and responsiveness to treatment. In recent times, there has been a growing utilization of nanoparticle (NP)-based detection strategies to create quick and efficient detection assays. Every single one of these platforms leverages the distinct characteristics of NPs to identify periodontitis. Plasmonic NPs include metal NPs, quantum dots (QDs), carbon base NPs, and nanozymes, exceptionally potent light absorbers and scatterers. These find application in labeling, surface-enhanced spectroscopy, and color-changing sensors. Fluorescent NPs function as photostable and sensitive instruments capable of labeling various biological targets. This article presents a comprehensive summary of the latest developments in the effective utilization of various NPs to detect periodontitis.

**Graphical Abstract:**

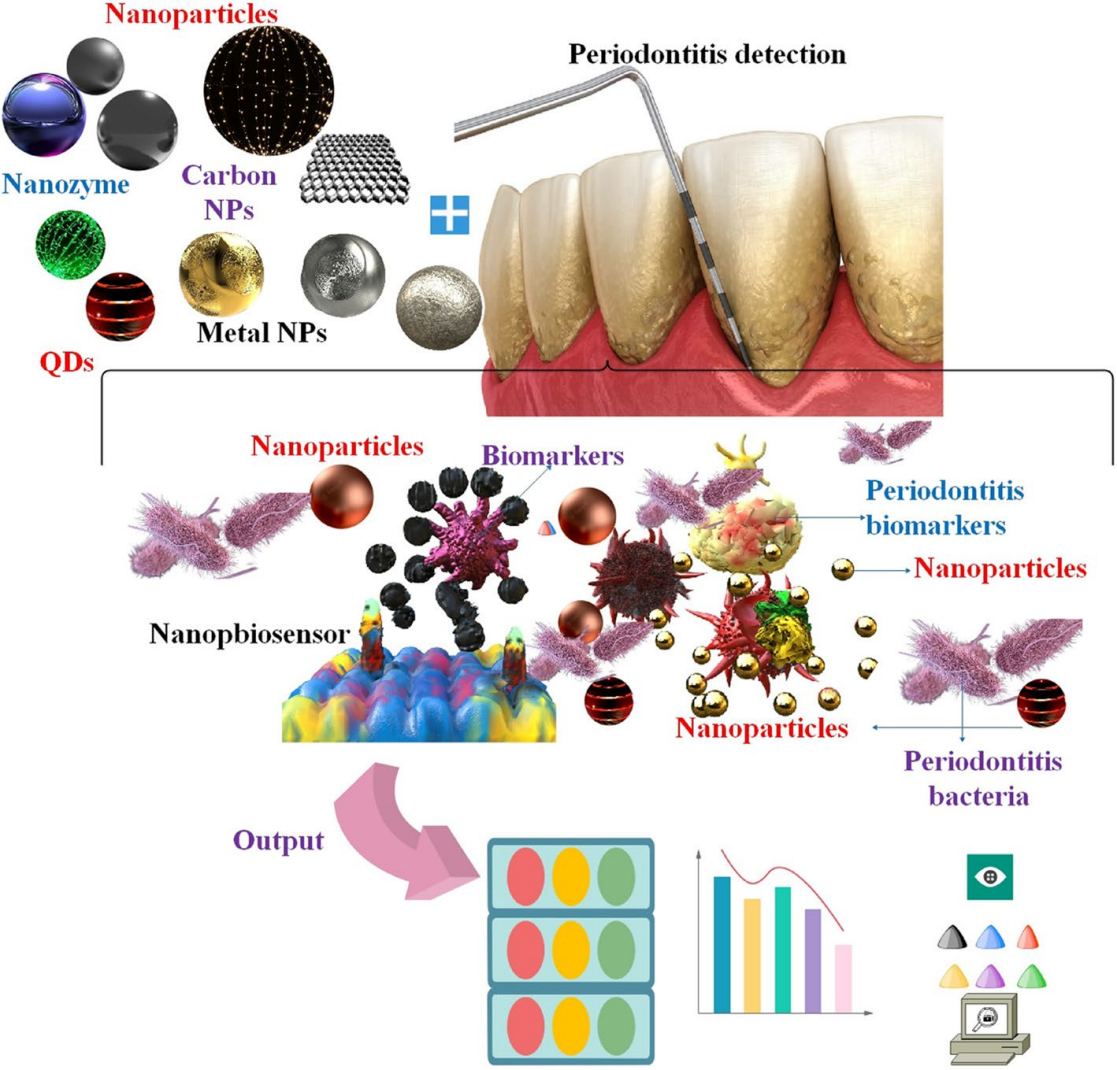

## Introduction

Periodontitis is a persistent inflammation characterized by bacterial-induced, host-controlled inflammation that causes harm to the soft tissue attachment and leads to the loss of the bone around the teeth [1, 2]. The development of periodontitis is characterized by the first establishment of bacterial communities that create a biofilm on the surfaces of the teeth. The relationship between the periodontal bacteria that trigger dysbiosis and the subsequent development of periodontal disease (PD) is complex [3]. Chronic hyperglycemia, suboptimal oral hygiene, smoking, genetic predisposition, hormonal imbalance, and systemic complications are all risk factors for periodontitis [4]. In addition, various inflammatory illnesses known as PDs impact the gingiva, bones, and ligaments that support teeth. These problems may cause inflammation throughout the body and can lead to tooth loss [5]. Gingivitis is distinguished by gingival inflammation without bone or connective tissue loss. Gingivitis, while an essential precursor to periodontitis, does not invariably result in the development of the condition that impacts an estimated 7–15% of adults in Western societies [6].

In addition, according to the World Health Organization (WHO) report, it is estimated that severe PDs affect about 19% of the world's adult population, representing more than 1 billion cases worldwide. The main risk factors for PD are poor oral hygiene and tobacco use [7, 8]. The most prevalent oral illness in the gestational period, affecting 40–70% of individuals, is gingivitis, an early stage of PD [9]. Age-related disparities in PD are observed, and the condition progressively deteriorates. An epidemiological inquiry revealed that older people exhibited the highest incidence of chronic periodontitis (82%), with adults and adolescents following suit with 73% and 59%, respectively [10]. Periodontitis is characterized by gradual degradation of the alveolar bone around the teeth. If not treated, it may result in tooth mobility and eventual tooth loss. Epidemiological studies have shown a correlation between periodontitis and various systemic illnesses, including atherosclerosis, cardiovascular disorders, and rheumatoid arthritis [11, 12].

The diagnosis of periodontitis is determined through a comprehensive assessment of the patient's periodontal health. This includes evaluating the full-mouth plaque score, full-mouth bleeding score, probing depth, clinical attachment level, bleeding on probing (BoP), recessions, mobility, and migration. These evaluations provide a comprehensive understanding of the periodontal conditions specific to an individual patient [13].

Nevertheless, these approaches cannot identify the pathogens implicated [14, 15]. To develop tailored and effective treatment regimens for periodontitis, it is essential to accurately and promptly diagnose the current circumstances of the disease, including its existence, severity, and activity [16]. However, it cannot be obtained by traditional methods of clinical evaluation. Therefore, it is essential to devise a novel approach to promptly and precisely identify periodontitis problems and their advancement [17]. The quantity of the analyte in the reaction determines the signal that a biosensor produces in response to a chemical or biological reaction. Biosensors are useful in many fields, such as pharmaceutical research, disease monitoring, and detecting pollutants, pathogens, and disease-indicating substances in physiological fluids (blood, urine, saliva, sweat, etc.) [18, 19]. In recent years, the focus has been on biosensors that facilitate health surveillance, prevention, and treatment in real time. As the popularity of smart wearable devices has increased, they can now be used to predict disease, and it will become fashionable for individuals to improve their health in ways other than exercise by utilizing such devices [20]. The components used for biological detection, such as enzymes, antibodies, and nucleic acids, are tightly linked to transducers, such as optical, electrochemical, and piezoelectric devices. This connection adds complexity and allows for a more precise and quantitative understanding of biodegradation [21, 22]. Timely detection is crucial for effective management of periodontitis [23]. Numerous oral fluid indicators have been studied to diagnose PD, including host-derived proteins (e.g., immunoglobulins and enzymes), host cells (e.g., PMNs), bacteria and their byproducts, ions, hormones, and volatile compounds [24]. Incorporating nanostructured materials into bioassay methodologies has emerged as an area of rapid advancement. Many nanomaterials, such as nanotubes, nanofibers, thin coatings, nanorods, and NPs, have had their characteristics and uses delineated in the scientific literature [25, 26].

Graphene (GPH), silver nanoparticles (AgNPs), chitosan, hydroxyapaptite NPs, copper oxide (CuO), bioactive glass, mesoporous calcium silicate, titanium dioxide NPs, magnesium, calcium oxide, and iron compound are among the other NPs that have been covered. Because of their antibacterial properties, these NPs have found their way into a variety of dental materials, where they have proven useful in the identification and treatment of oral disorders as well as in the removal of smear layers and biofilms [27–29]. Exploring and employing nanotechnology in the battle against dental disorders, notably periodontitis, is crucial, especially in light of the worrying rise in antimicrobial resistance [30]. Conversely, periodontitis often has a less noticeable beginning, characterized by a gradual deterioration of tissue hence making it difficult to detect and anticipate its advancement in the early stages [31]. Different categories of NPs, such as fluorescent, magnetic, and metallic NPs, have been successfully used to detect infectious illnesses. Fluorescent NPs function as sensitive and durable probes capable of labeling several biological targets. Point-of-care testing (POCT) has transformed medical testing by enabling convenient tests to be performed near the patient's care location rather than being limited to a medical laboratory. This has proven particularly advantageous for poor nations with inadequate infrastructure since testing often entails dispatching samples to external facilities and enduring hours or days of waiting for the results. Nevertheless, the progress of POCT devices has encountered difficulties, with the essential requirements of simplicity, accuracy, and cost-effectiveness playing a crucial role in ensuring the viability of these tests. The significant factors driving POCT, especially in detecting infectious diseases, are microfluidics and lab-on-a-chip technologies [22, 32].

For example, variations in pro-inflammatory cytokines and proteases are useful as markers of the prevalence, severity, and development of PDs. The biomarkers garnered the most attention are matrix metalloproteinases-8 (MMP-8), IL-1β, and tumor necrosis factor-alpha (TNF-α). This is because these biomarkers are directly linked to the destruction of periodontal tissue, and their concentrations change as the destruction of periodontal tissue progresses. It has been shown that the simultaneous identification of many biomarkers increases the precision and effectiveness of the diagnosis of periodontitis and the assessment of the treatment response. Typically, these indicators are detected by measuring their levels in saliva or gingival crevicular fluid (GCF). Despite its benefits of substantial sample size and convenient collection, saliva primarily reflects the overall state of the whole mouth rather than particular individual teeth, limiting its specificity for detection purposes. By contrast, GCF emanates from the gingival tissues of certain teeth, enabling a more precise and reliable detection of PD. Nevertheless, the GCF volume is insufficient (often < 2 μL) for the analysis of numerous biomarkers, posing a challenge to accurately and simultaneously identify periodontitis biomarkers in GCF [33].

Among the most common nanomaterials used in these periodontitis detection nanobiosensors were metal NPs, magnetic NPs, carbon nanotubes, and quantum dots (QDs); these materials considerably enhanced the sensors' sensitivity and accuracy [34]. Using nanobiosensors to analyze periodontal biomarkers could be considered a quick and uncomplicated method for differentiating healthy patients from those with periodontitis. This review will concentrate on the advancements in research concerning the integration of NPs into biosensors to detect periodontitis. Consequently, an initial examination was conducted on the attributes of periodontitis and traditional diagnostic approaches. We then investigated the dental applications of NPs and nanobiosensors. In conclusion, we have examined the function of diverse categories of diagnostic NP-based nanobiosensors in the identification and diagnosis of periodontitis. Our objective is to forecast forthcoming periodontal infections by analyzing and forecasting current trends and diagnostic approaches associated with them, with the hope that this will lead to increased utilization of nanobiosensors in periodontitis research and the progression of nanodentistry dental development.

## Characteristics of periodontitis and pathogens

Periodontitis classification has been recently established into phases and grades based on the criteria set by the World Workshop on the Classification of Periodontal and Peri-Implant Diseases and Conditions [35]. Periodontitis is caused mainly by subgingival biofilms of structured bacterial populations [36]. *Pophyromonas gingivalis (P. gingivalis)* is a crucial anaerobic pathogen that plays a significant role in forming severe lesions. The objective of periodontal therapy is to inhibit the growth of biofilms below the gingiva line and to restore the balance of tissues [37]. Periodontal pathogens may consist of various bacteria, including Aggregatibacter actinomycetemcomitans (*A. actinomycetemcomitans)*, *Fusobacterium nucleatum* (*F. nucleatum*), *Peptostreptococcus micros*, *P. gingivalis*, *Prevotella intermedia*, *Treponema denticola*, *Treponema forsythia*, and potential periodontal pathogens like *Filifactor alocis* and *Parvimonas micra*. Mainly, organisms from the red complex (*P. gingivalis*, *T. forsythia*, and *T. denticola*) are considered significant. Additionally, there are at least 17 new candidate organisms, including species or phylotypes from various phyla. *Bacteroidetes*, *Candidatus Saccharibacteria*, *Firmicutes*, *Proteobacteria*, *Spirochaetes*, Synergizes, and maybe Archaea from the domain, have been linked to PD [38].

As the condition worsens, there is a possibility of tooth movement and eventual tooth loss. Quantifying the occurrence of periodontitis is challenging due to the inconsistent criteria used to define cases. Periodontitis may be classified based on its scope (localized or widespread) and intensity (mild, moderate, or severe) in most cases [39]. The primary categories of periodontitis include chronic periodontitis, aggressive periodontitis, periodontitis as a symptom of systemic disorders, necrotizing ulcerative periodontitis, abscesses of the periodontium, and periodontitis linked with endodontic lesions [40].

Furthermore, as the condition advances, it leads to significant morbidity characterized by the development of periodontal abscesses and tooth loss, and in the later stages, the experience of pain [41]. The severity of the sickness primarily relies on the bacterial components and the host's reaction. During the early phase of the illness, inflammation is confined to the gingiva (gingivitis). Subsequently, it progresses to affect the underlying tissues, resulting in swelling of the gingiva, bleeding, and bad breath (halitosis). During the advanced stage of the illness, the collagen that supports the periodontium starts to deteriorate, resulting in the resorption of the alveolar bone. Additionally, the epithelial tissue of the gingiva migrates, leading to the development of pockets [42].

**Fig. 1:**
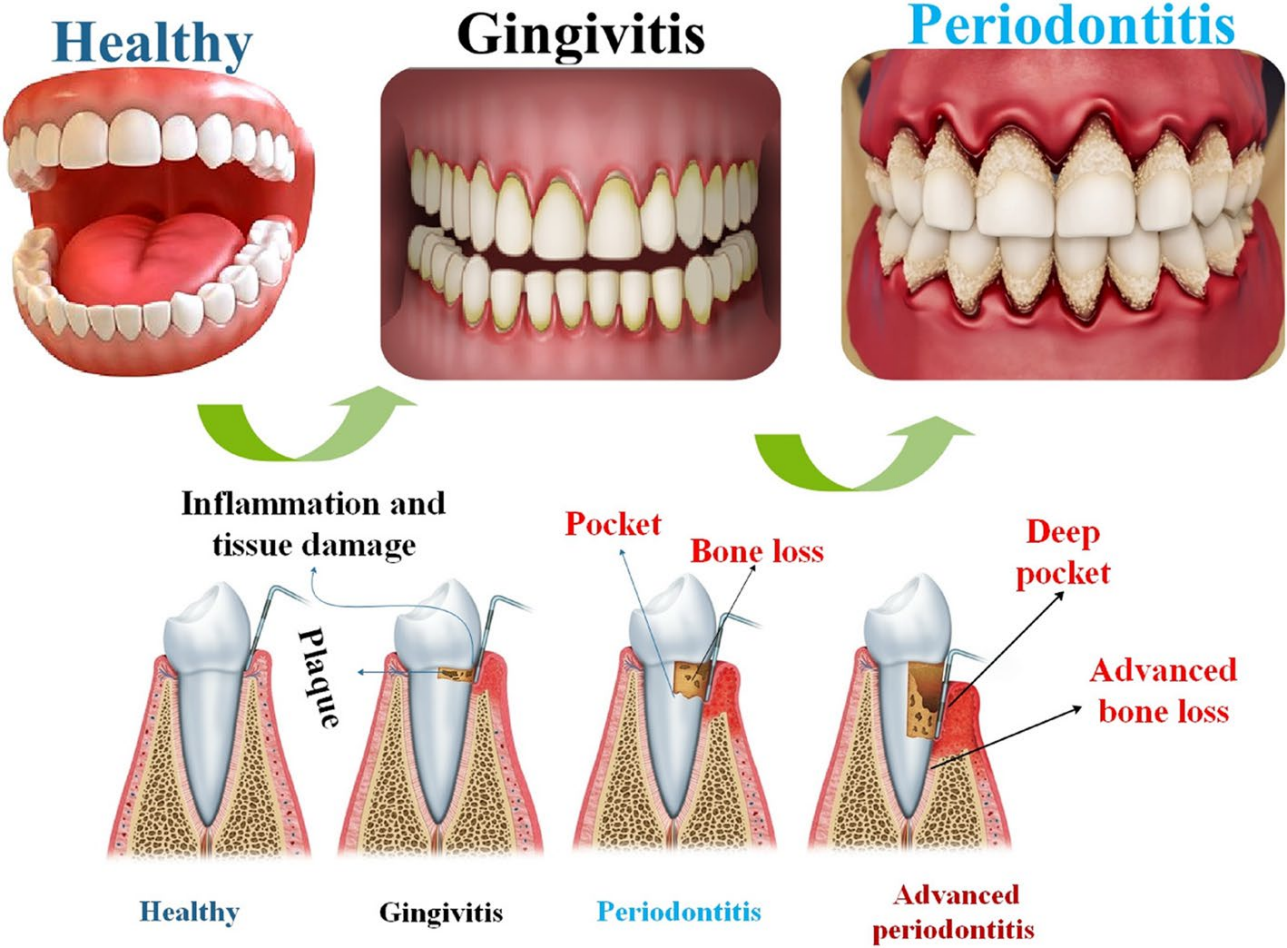
Bone and tissue loss in the gingiva and around the teeth is a hallmark of periodontal disease. Gingiva recession and tooth loss are among the several consequences that might result from this. Stages of periodontal tissue disease may be categorized according to severity and progression: gingivitis (the first stage), early periodontitis (the second stage), and advanced periodontitis (the third stage)

The clinical periodontal diagnostic in maintenance patients aims to assess and track the likelihood of PD progression. During every recall session, it is crucial to consistently examine the risk of advancement at the patient, tooth, and site levels [43]. At the individual patient level, the relevance lies in systemic disorders, cigarette smoking, adherence to the recall program, decline in support as the patient ages, high scores for plaque and/or bleeding throughout the mouth, and the incidence of residual pockets [44] (Fig. 1).

### Common methods of diagnosing periodontitis

Periodontal evaluation should be a standard component of oral examination for all patients. In addition to being done routinely as part of continuing oral health treatment, periodontal screening should be done for all new patients utilizing techniques such as the Basic Periodontal Examination/Community Periodontal Index (CPI) or Periodontal Screening Record. This entails documenting data from whole mouth probing and bleeding as well as evaluating other pertinent factors such as plaque concentrations, involvement of the furcation, recession, and tooth movement. The radiographic evaluation of alveolar bone levels is determined by the specific clinical circumstances and is necessary to evaluate the extent of bone loss in individuals with periodontitis. Conducting a thorough evaluation of risks, such as the presence of diabetes and smoking habits, and implementing strategies to mitigate those risks, such as encouraging smoking cessation, should be a fundamental part of periodontal care [45].By utilizing a blunt, standardized probe to examine each tooth for bone loss, a reliable diagnosis of periodontitis may be made [46]. However, visual analysis of these indicators by dentists with expertise is necessary, and there is a greater risk of misdiagnosis. Later, cone-beam computer tomography (CBCT), computer tomography (CT), fiber optic trans-illumination (FOTI), quantitative light-induced fluorescence (QLF), and intraoral periapical and bitewing radiographs were among the auxiliary methods used for therapeutic purposes [47, 48].

**Fig. 2:**
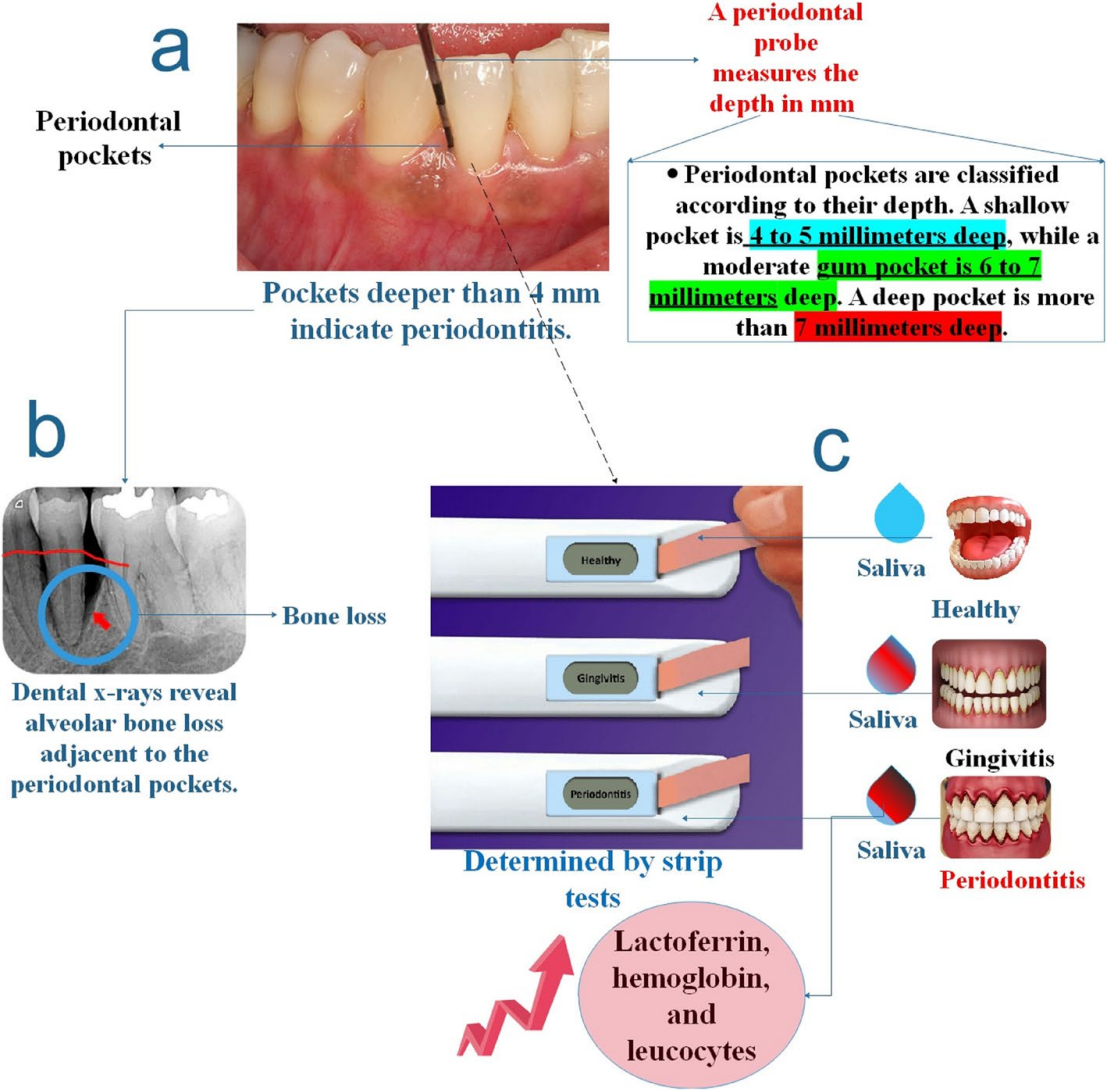
**a** An examination of the teeth and periodontal tissues, together with the use of a probe to determine the depth of the pockets, is typically sufficient to make a diagnosis. Periodontitis is characterized by pockets deeper than 4 mm. **b** Dental X-rays reveal alveolar bone loss around the periodontal pockets. Periodontal biomarker analysis using test strips may be a rapid and straightforward way to distinguish between persons with periodontitis and those in normal dental health. The increase in lactoferrin, hemoglobin, and leucocytes revealed by strip testing may give a non-invasive method of identifying periodontal disease [48, 50]

Furthermore, while existing clinical diagnostic methods for periodontitis effectively evaluate its extent and previous periodontal damage, they fail to indicate the disease's present state or facilitate its progression prediction or monitoring. On the other hand, biomarkers that are linked to periodontitis have the potential to offer insights into the current disease status, evaluate the efficacy of treatment, and forecast forthcoming hazards. Within twenty minutes, biomarkers of periodontitis can be identified directly at the site of examination using POCT. The product's cost-effectiveness, ease of use, and effectiveness could motivate people to prioritize their oral health and decrease the necessity for dental interventions [49] (Fig. 2).

#### Clinical examination and periodontal probing

The gingival and periodontal tissues ought to be examined sequentially. The initial step taken by most operators is a visual examination of the gingival tissues, which involves a subjective assessment of the degree of swelling and color of the tissues to determine whether gingival inflammation is present or absent. Additionally, this visual inspection evaluates the level of oral hygiene by quantifying plaque and calculus. The evaluation of probing depths then ensues. Selecting a periodontal probe is the initial determination that must be made [51]. Periodontal probing, radiographic analysis, gingival index, mobility charting, and assessment of the quantity of connected gingiva are all components of a periodontal examination. Simple instruments and little clinical calibration on the examiner's side are needed for these clinical tasks. Nonetheless, a general exam that determines the location of caries, the plaque index, and the patient's medical and dental history is still necessary in addition to these exercises. When it comes to identifying PD and assessing treatment, probing is still considered to be the most crucial testing technique. However, when paired with information from a radiographic study and gingival index, probing has a far more profound significance. A gingival index should be an essential component of a regular examination as the relevance of the pocket depth relies not only on the height of the alveolar bone but also on the level of gingival inflammation. In addition to probing, a radiographic examination helps determine the architecture of a bone defect and provides information on the length, shape, and breadth of the periodontal ligament gap. However, without information on mobility patterns, gingival border location, and pocket depths, radiographic analysis is essentially useless. In the last ten years, several advanced instruments and indices for periodontal exams have been developed. Sadly, many of these new tools are impractical for use in clinical settings in their current configurations. Future developments will undoubtedly focus on making these tools and indices simpler so that regular dentists and specialists alike may more readily apply them to the clinical practice of periodontics [52]. Additionally, in patients receiving periodontal maintenance therapy, prolonged bleeding during probing at subsequent maintenance appointments is a significant predictor of continued disease progression risk [53, 54].

In epidemiological research, the CPI investigation of the WHO may be utilized to designate a score for each sextant, with the score being determined by the site that is most significantly affected. With a 0.5 mm ball tip (to reduce the probe's penetration into soft tissues and aid in calculus identification), a black band between 3.5 and 5.5 mm, and rings at 8.5 and 11.5 mm, the WHO CPI probe is mainly designed for this use. More specific data, especially for individuals with periodontitis, could be needed for each patient in a clinical setting to document the exact depths of probing used across the dentition. For this reason, a range of periodontal probes are available, including computerized periodontal probes (like Florida probes) and manual probes (like Williams, UNC PCP-15) [45, 55]. In addition, when inflammation is absent, the probe tip fails to reach the junctional epithelium at its base [56]. Furthermore, the presence of persistent BOP at locations that also exhibit rising probing depths is a robust indication of the likelihood of illness development in the future [57].

#### Radiographic assessment

Radiography is primarily influenced by and subservient to the findings of the clinical examination. Hence, it is advisable to use existing radiographs, obtained for other reasons such as caries diagnosis, if feasible, to assist in evaluating alveolar bone levels [58]. For improved identification of changes in alveolar bone levels, it is advisable to adopt paralleling methods for intraoral periapical and ensure that consecutive radiographs are positioned consistently across time [59]. When utilizing contemporary panoramic machines, the radiation dosage is lower when taking a panoramic radiograph with a minimal number of additional periapical radiographs, based on the clinical need, compared to taking a full-mouth series of periapical radiographs. Moreover, the picture quality provided by contemporary panoramic machines is so high that there is no need for any supplementary periapical radiographs [45]. From a clinical standpoint, the assessment of periodontal health may be accomplished by assessing the clinical attachment loss (CAL) by probing pocket depths and gingival recession. Nevertheless, this technique is constrained by its limited dependability on the force of probing, the angle of approach, the positioning, and the diameter of the tip [60, 61]. If the CAL is inaccessible, radiographic bone loss (RBL) should be used. Computer-aided diagnosis (CAD) has been used for the detection of cavities, periodontitis lesions, maxillary sinusitis, osteoporosis, and other diseases in the area of oral and maxillofacial medicine [62]. Using computer-based methods for acquiring and processing images will enhance the significance of radiography in periodontal diagnostics. Subtraction radiography, which relies on digital pictures, may effectively enhance the visibility of temporal changes in lesions [63].

## Nanomaterials used in dental diagnostic applications

When selecting NPs for application in the domain of nano dentistry, consideration is given to their chemical, physical, and biological characteristics and nanostructures. Nanostructures are utilized in dental diagnostics and innovations [64].

Currently, the focus of illness diagnostics at the molecular level is centered on the latest advancements in nanotechnology. The distinct electrical, magnetic, luminescent, and catalytic characteristics of NPs are advantageous for swiftly, sensitively, and efficiently detecting microbial agents and surmounting drug resistance [65].

The researchers have successfully created a kind of NP called hafnium oxide nanoparticles (Hf PS NPs), made up of a combination of polymeric silane and hafnium oxide. These NPs have intrinsic therapeutic properties. A demonstration was conducted to show the use of a high-affinity pathogen-selective peptide for molecularly targeted X-ray imaging of the cariogenic pathogen *S. mutans*. Experiments conducted outside a living organism, using human tooth samples, showed a significant difference in X-ray absorption between NPs and tooth material. This work is the first study to showcase the use of HfO_2_-based NPs for both diagnostic and antibacterial therapy, eliminating the need for supplementary medication [66].

Researchers focus on developing innovative bio-based NPs for the precise detection of active caries in vitro. The NPs consist of a cationic fluorescein-labeled starch that is safe for consumption. These NPs are designed to emit fluorescence when exposed to a typical dental curing light. Additionally, they are programmed to break down into harmless substances in the mouth after they have identified a carious lesion that requires treatment. When human teeth are exposed to cationic fluorescent NPs with a positive charge of + 5.8 ± 1.2 mV and a size of 101 ± 56 nm, these NPs preferentially light up areas of active tooth decay but do not illuminate the healthy surface of the tooth. These innovative NPs provide a distinctive approach to aid in the early detection of active carious lesions, which has the potential to influence dental therapy directly py [67].

Photonics approaches have used core–shell nanostructures in several applications for diagnosis and treatment. This study presents a new core–shell nanostructure design that functions as a contrast agent for dental adhesion assessment using multimodal optical imaging. The nanostructure is composed of a core particle made of rare-earth-doped (NaYF4:Yb 60%, Tm 0.5%)/NaYF_4_, which has a hexagonal prism shape with a base side length of around 51 nm. The core is surrounded by a shell made of the highly refractive substance TiO_2_, which has a thickness of about 15 nm. Investigators demonstrated that the TiO_2_ shell improves the visibility in optical coherence tomography (OCT). At the same time, the rare-earth-doped core changes the excitation light from 975 nm to an emission with a peak at 800 nm for photoluminescence imaging. This NP core–shell contrast agent was used to showcase the OCT and photoluminescence wide-field photographs of a human tooth. Furthermore, the core–shell NPs (CSNps) mentioned were evenly distributed in the primer of a readily accessible dental bonding system, allowing the distinct visualization of dental adhesive layers using OCT. Furthermore, their findings emphasize that the upconversion photoluminescence in the NIR band is well-suited for imaging deep dental tissue [68].

## Biosensors in the field of dentistry

A biosensor is an analytical instrument that combines a biologically active component with a suitable physical transducer to produce a quantifiable signal that is directly proportional to the concentration of chemical substances in any kind of sample [69]. Biosensors are compact, self-contained, analytical, integrated scientific instruments utilized for the identification and quantification of target substances [70]. The complex and quantitative nature of biodegradation arises from the close association between biological detection components (such as antibodies, enzymes, and nucleic acids) and transducers (such as piezoelectric, optical, and electrochemical devices) [71]. The intensity of the output signal typically corresponds to a collection of analyzers. In conclusion, the outcomes are generated by using implemented devices and the programming framework at play. These provide a more user-friendly and sophisticated visualization that even non-specialists can operate [72].

**Fig. 3:**
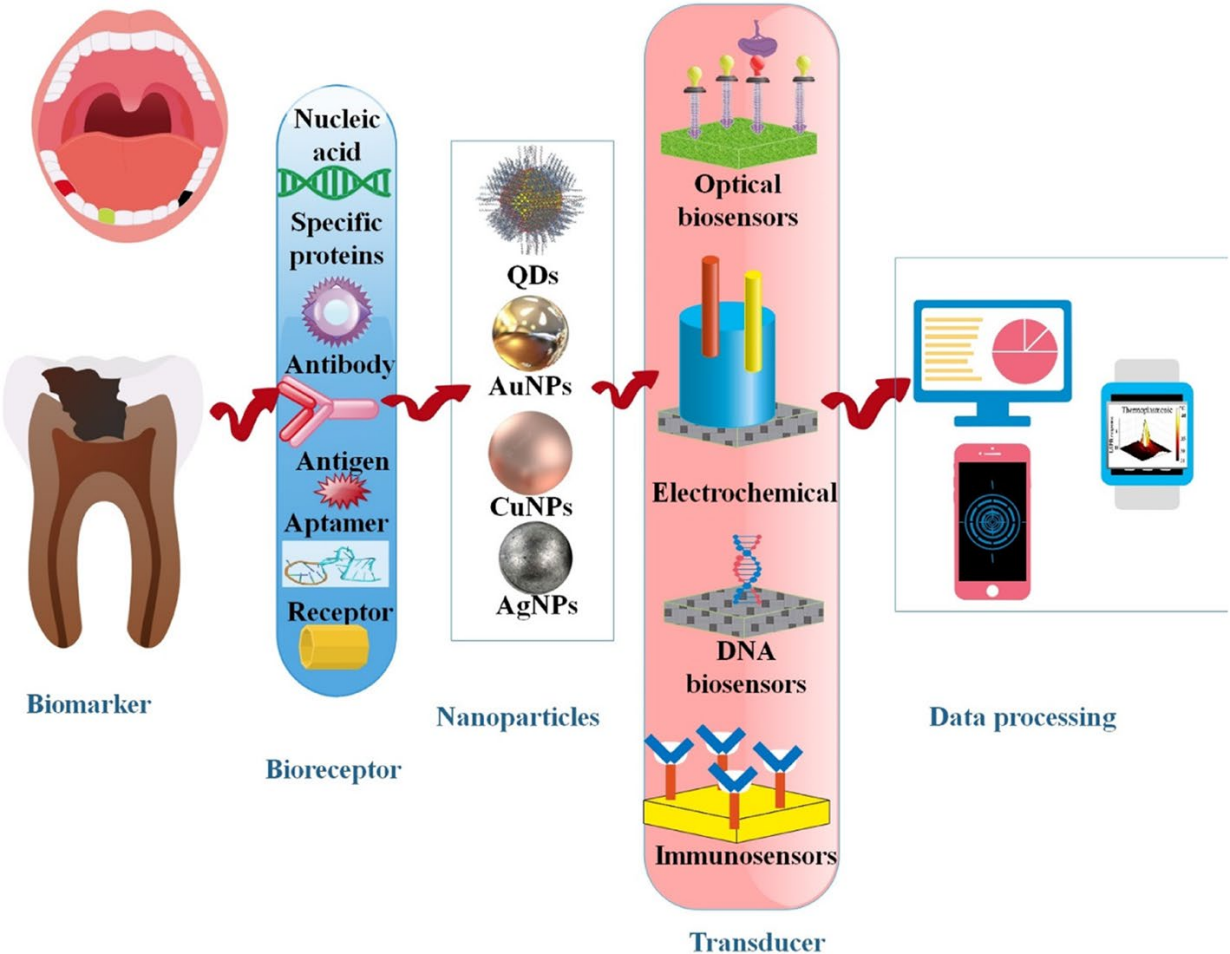
Periodontitis may be detected using various platforms, including those based on electricity, DNA, immunosensors, electrochemistry, and optics. Nanomaterials are often used as transducer materials, playing a crucial role in advancing biosensors. A biosensor has four essential components: a bioreceptor, a transducer, a signal processor for transforming electrical signals into the required form, and an interface for displaying the results. Various metal nanoparticles (NPs), including AuNPs and AgNPs, as well as diverse forms of graphene oxide, carbon dots, and quantum dots, are synthesized using easy techniques and serve as distinct nanoplatforms for detecting circulating biomarkers

Metal NPs, MNPs, carbon nanotubes, and QDs are among the most notable nanomaterials utilized in these nanosensors; they all contributed significantly to their increased sensitivity and precision [34]. More researchers have been interested in electrochemical biosensors in the last several decades than in other analytical methods like chromatography, spectrophotometry, fluorescence, migration techniques, or flow systems. These technologies may be used with labs-on-chips to provide superior POC analytical platforms because of their sensitivity, speed, and practicality. Their one-of-a-kind properties make them great instruments for a wide variety of analytes, including medications, proteins, markers, microbes, viruses, and bacteria [73]. For the immunomagnetic separation of proteins, viruses, and nucleic acids, MNPs coupled with antibodies have also been used. Synthesis parameters like polymer addition time and temperature, specific capping agents, and surface modification can all modulate the shape and magnetic properties of MNPs. This allows for the addition of functional groups for attaching various ligands, such as antibodies, proteins, and nucleic acids, which can then be used for target identification and quantification [74, 75] (Fig. 3).

Furthermore, a crucial prerequisite for nanobiosensors is the presence of immobilization methods that may be used to fix the bioreceptor in place, hence enhancing the feasibility and efficiency of its interaction with the bioanalyte [76]. Nanobiosensors are small, nanoscale devices that can detect biomolecules in saliva that are linked to many types of oral disorders. For example, biosensors can detect fungal or bacterial infections very rapidly, which helps dentists diagnose and treat these problems more easily [70]. Undoubtedly, nanodentistry offers several advantages compared to traditional systems, including enhanced bio-regeneration, significant antimicrobial effects resulting from anti-biofilm properties, improved hardness of composites, and better sealing of fillers. However, the clinical exploration of nanodentistry is limited due to factors such as high cost, precise placement requirements, potential toxicity, expensive development process, and international regulations [77, 78]. PD may be diagnosed using biosensors on a nanoscale. These sensors can detect chemicals in saliva, blood, and GCF, among other physiological fluids [79]. Furthermore, a crucial prerequisite for nanobiosensors is the presence of immobilization methods that may be used to fix the bioreceptor in place, enhancing the feasibility and efficiency of its interaction with the bioanalyte [76].

Owing to the limited awareness of dental conditions and insufficient availability of healthcare services, many individuals worldwide have not received prompt medical intervention or have been neglected altogether [80]. The implementation of dental peripheral sensors has the potential to enhance the efficacy of dental procedures while substantially mitigating pain and complications [81]. The widespread use of dental wearable sensors may help address the pressing need for dental services without imposing extra cost constraints. Dental wearable sensors have the potential to alleviate the financial strain on governments in the healthcare sector and enhance the general well-being of the people. Currently, some dental sensors have been documented to monitor dental caries, orthodontic therapy, and dental implants [82]. In periodontitis, proteases play a crucial role as virulence agents. Gingipains, a kind of protease, are prevalent in PD and show great promise as a biomarker because of their role in the development and advancement of the illness. Creating an innovative, ultrasensitive silicon nanopore protease activity sensor that does not need labels and can identify gingipain activity at clinically significant doses. Researchers have shown that the solid-state nanopore-biosensor can detect trypsin with a limit of detection (LOD) of 0.005 ng/mL (0.2 pM). Subsequently, the detection technique that was created for the model enzyme was used for the detection of gingipains. The LOD for detecting gingipains was determined to be 1 ng/mL (0.02 nM). At a concentration of 0.1 µg/mL, the signal showed a recovery rate of 27%. These findings demonstrate that the sensitivity and dynamic range of the assay are suitable for the clinical diagnosis of periodontitis [83].

Adaptive danger detection and health quality monitoring might be affected by nanosensors directly interfaced with biomaterials, according to another research. Because of its nanoscale structure, GPH can detect analytes with a great degree of sensitivity. Scientists have shown here that water-soluble silk can be produced using GPH. As a result, biomaterials like tooth enamel may undergo the close biotransfer of GPH nanosensors. An adaptable sensing platform with comprehensive biointerfaces for detecting specific analytes is the end product. Researchers demonstrate bioselective detection of bacteria at single-cell levels, for instance, by self-assembling antimicrobial peptides onto GPH. By using a resonant coil, the need for onboard power and external connections is rendered obsolete. As a whole, this method of connecting GPH nanosensors to biomaterials offers a flexible way to detect biochemical targets anywhere [84].

The noninvasive diagnosis of *Helicobacter pylori* (*H. pylori*) infection is very appealing in a separate research investigation. This work examined the process of obtaining single-strand DNA (ssDNA) from *H. pylori* in dental plaque, and the combination of a previously designed 43-mer *H. pylori* DNA biosensor with the acquired target ssDNA (tDNA). The biosensor has a LOD of 12 fM dsDNA. At 100% sensitivity and 97% specificity, the dental plaque detection findings were in excellent agreement with the urea breath test (UBT) results. Researchers findings suggest a strong correlation between *H. pylori* residing in dental plaque and stomach *H. pylori* infection. This DNA biosensor shows excellent promise for noninvasively diagnosing *H. pylori* infection by detecting dental plaque samples [85].

This work developed a new portable diagnostic biosensor that may detect periodontitis by using two common inflammatory salivary biomarkers, Human Neutrophil Elastase (HNE) and Cathepsin-G. The biosensing technology relied on quantifying proteolytic activity using targeted protease probes. These probes are composed of protease substrates that are chemically attached to a magnetic bead on one end and to the Au sensor surface on the other end. When undamaged, this causes the golden sensor to appear black. After undergoing proteolysis, the fragmented magnetic beads will be drawn towards an external magnet, therefore exposing the sensor surface's golden hue, which may be easily seen without the use of any instruments. The biosensor demonstrated the ability to accurately and precisely detect HNE and Cathepsin-G in both liquid solution and saliva samples, with a minimum detectable concentration of 1 pg/mL for HNE and 100 fg/mL for Cathepsin-G. The analysis of samples from patients with periodontitis and a healthy control demonstrated the capability of the multiplex biosensor to identify the existence of HNE and Cathepsin-G activity directly at the site. This technique is expected to be a valuable biochip array that can be easily used in affordable POC devices [86].

A non-enzymatic and highly electrocatalytic H_2_O_2_ biosensor was developed in this research endeavor by utilizing an innovative electrode consisting of chitosan, black phosphorus nanosheets (BP NSs), and CuO NPs. The combined utilization of CuO and BP exhibited exceptional electrocatalytic capabilities, characterized by a low practical detection limit of 30 nM, remarkable sensitivity of 138.00 μA mM^−1^ cm^−2^, excellent selectivity, remarkable reusability, and long-term stability. This biosensor detected H_2_O_2_ levels in saliva and GCF samples, allowing for accurate differentiation between patients with periodontitis and healthy individuals. At the cellular level, this device was effectively used to detect the release of H_2_O_2_ from macrophages and gingival fibroblasts [87].

A combination of nanomaterials and artificial intelligence is presently being utilized to accomplish multiplexing. Continually underway is the development of low-cost POC devices for use in developing nations. Subsequently, enhanced and novel NP-based platforms have been developed to identify biomarkers associated with infectious and non-infectious diseases. These advancements have resulted in diagnostic procedures that are more precise and effective. The consolidation of intricate processes onto a solitary apparatus has enabled the detection of diseases in real time, even in regions where resources are scarce [75].

## Use of biomarkers to diagnose periodontitis

Oral and PD diagnostic research is progressing towards approaches that can identify and assess periodontal risk using objective metrics like biomarkers. A biomarker is a material that serves as an indicator of a biological condition and provides an objective measurement for assessing both current and future disease activity. A biomarker is a material that is quantitatively examined and assessed as an indication of normal biological activities, pathological processes, or the pharmacological effects of a therapeutic intervention. Saliva, serum, and GCF are used as biological media to identify biomarkers in periodontal health and disease. A solitary biomarker will be insufficient to forecast the activity and severity of PD. Biomarker combinations are used to predict the activity of the illness [88]. Extensive research has been conducted on the potential of GCF to release host response factors. It comprises an amalgamation of biofilm constituents derived from blood, the host tissue, and plaques, including enzymes, bacterial antigens, small molecules, proteins, cytokines, antibodies, and bacterial antigens. Biomarkers for periodontal infection also consist of proteomic, genetic, and microbial components, among others [89]. Proteomic biomarkers include lactoferrin, translactoferin, aminopeptidase, essential phosphatase, IgM, MMP-13, MMP-8, and MMP-9. Hereditary biomarkers include polymorphisms in IL-1, IL-10, tumor rot factor, and others. Microbiological biomarkers include *Aggregatibacter actinomycete mcomitans*, *Campylobacter rectus*, *Mycoplasmas*, *P. gingivalis*, *Prevotella intermedia*, and *Peptostreptococcus*. There are several biomarkers, including calcium, cortisol, hydrogen sulfide, methyl mercaptan, and pyridine [90].

As oral microbes adjust to environmental variations in their habitats within the mouth, proteomics provides a novel method for comprehending the alterations. Periodontitis has been observed to induce downregulation of proteins in addition to upregulation. Protease-inhibiting properties of cystatin include lysosomal cathepsins B, H, and L, which may be implicated in periodontal tissue degradation. The control group exhibited a greater abundance of cystatin than the periodontitis group [91, 92]. Moreover, immunoglobulins (Ig) serve as critical specialized defense mechanisms within the saliva. Secretory IgA (sIgA), which is generated by plasma cells located in the salivary ducts, is the most abundant form of Ig found in saliva. Less IgG and IgM are present in the saliva. IgA, IgG, and IgM influence the oral microbiota through metabolic inhibition or by inhibiting bacterial adhesion. A multitude of investigations have been undertaken to ascertain whether salivary sIgA levels are correlated with different types of PD. A positive correlation was observed between the concentration of IgA and the degree of inflammation [24]. The researchers' objective was to assess the efficacy of diagnostic salivary tests in determining the periodontal condition. Saliva samples, both stimulated and unstimulated, were collected from twenty individuals who were healthy and twenty individuals who had stage III grade B generalized periodontitis. The samples were analyzed for lactoferrin, alkaline phosphatase (ALP), calcium levels, density, osmolarity, pH, phosphate levels, buffer capacity, salivary flow rate, and dynamic viscosity. A semi-quantitative urine strip test was used to assess indicators of inflammation in saliva, including erythrocytes, leukocytes, urobilinogen, nitrite, glucose, bilirubin, and ketones. Additionally, clinical periodontal parameters and pathogenic microorganisms were evaluated. The levels of lactoferrin, hemoglobin, and leukocytes were substantially elevated in both stimulated and unstimulated saliva of patients with periodontitis compared to healthy individuals. Additionally, the levels of ALP were more significant in the unstimulated saliva of periodontitis patients. Periodontal biomarker analysis with test strips may be regarded as a speedy and effortless method for differentiating between individuals with periodontitis and those in a healthy state. The rise in lactoferrin, hemoglobin, and leucocytes, as detected by strip testing, might potentially provide a non-intrusive approach for diagnosing periodontal conditions [55].

Periodontists may find biomarkers to provide periodontal patients early warning, prognosis, and treatment information by using chair-side diagnostic tests that use entire saliva. These biomarkers' sensitivity and specificity are crucial in determining their diagnostic validity. Meta-analyses and literature reviews have recently placed a great deal of emphasis on five host-derived biomarkers: MMP-8, Hemoglobin (HB), MIP-1α (Macrophage inflammatory protein-1 alpha), MIP-1γ, and IL-1β. These biomarkers can potentially be used as early detection indications for periodontitis. Chair-side Lab-on-a-chip (LOC) technology has the potential to significantly improve periodontal health globally by aiding in the identification of biomarkers in saliva.[93]. Out of the several salivary biomarkers evaluated, researchers determined that IL-1β, MMP-8, and MMP-9 could identify people with periodontitis. Furthermore, IL-1β and MMP-8 hybridization may be able to differentiate between those who have gingivitis and those who do not [94]. The gingivitis group exhibited significantly increased levels of MIP-1α and Prostaglandin E2 (PGE2) (2.8-fold) compared to the control group. After undergoing dental prophylaxis, there was no statistically significant reduction in the average levels of biomarkers in the gingivitis group compared to their initial levels. Nevertheless, the levels of IL-1β, IL-6, and MMP-8 approached those observed in individuals who were in good health. Conversely, the levels of PGE2 and MIP-1α remained considerably elevated in the group with gingivitis as opposed to the control group. The likelihood ratio studies provided evidence that PGE2 concentrations, when used alone or in combination with MIP-1α, could accurately distinguish between gingivitis and a normal state. PGE2 and macrophage inflammatory protein-1 alpha (MIP-1α) concentrations in the saliva may serve as determining factors between individuals with gingivitis and those with unaffected gums. In addition, for several weeks after regaining health following dental prophylaxis, gingivitis patients continue to produce elevated concentrations of inflammatory mediators in their saliva [95].

Subgingival plaque contains almost 600 distinct bacterial species, which contribute to the development of PD. *Treponema denticola*, *P. gingivalis*, and *Tanerella forsythia* are the primary microorganisms responsible for causing PDs. The "red complex" is mainly associated with PD. *Actinobacillus actinomycetemcomitans* is a species linked to chronic, early onset, and aggressive periodontitis. Additional infections, including *C. ochracea*, *E. corrodens*, *C. recta*, and *F. nucleatum*, have also been linked to chronic periodontitis [96, 97].

MicroRNAs (miRNAs) are a group of small, single-stranded, naturally occurring, well-preserved, non-coding RNAs that play crucial roles in regulating a wide range of cellular and physiological processes. These processes include cell growth, specialization, cancer development, and tissue repair [98, 99]. It is well understood that miRNAs control transcription, messenger RNA stability, and protein translation. Their significance in the field of dental stem cell biology has been established, as they exert an impact on various processes, including dental stem cell differentiation, immune response, apoptosis, and inflammation of the dental pulp. Multiple bacterial components and dental detritus have contributed to the dysregulation of these indicators. It has demonstrated considerable utility as a diagnostic and prognostic indicator in PD. Based on the results obtained from a study, it can be concluded that saliva-derived miRNA-146a and miRNA-155 have the potential to function as reliable and non-intrusive biomarkers in the diagnosis and prognosis of PD among individuals with and without diabetes [100, 101].

Higher salivary cortisol levels were found in those with severe periodontitis, according to research that assessed the relationship between PD and stress, distress, and coping strategies. An additional investigation was carried out to examine variations in salivary calcium levels between people with periodontitis and those who were periodontally healthy. Researchers indicate that individuals with periodontitis tend to have higher salivary calcium concentrations, as shown by the fact that those in the high calcium salivary group had far more undamaged teeth than their counterparts in the low calcium salivary group [102].

**Fig. 4:**
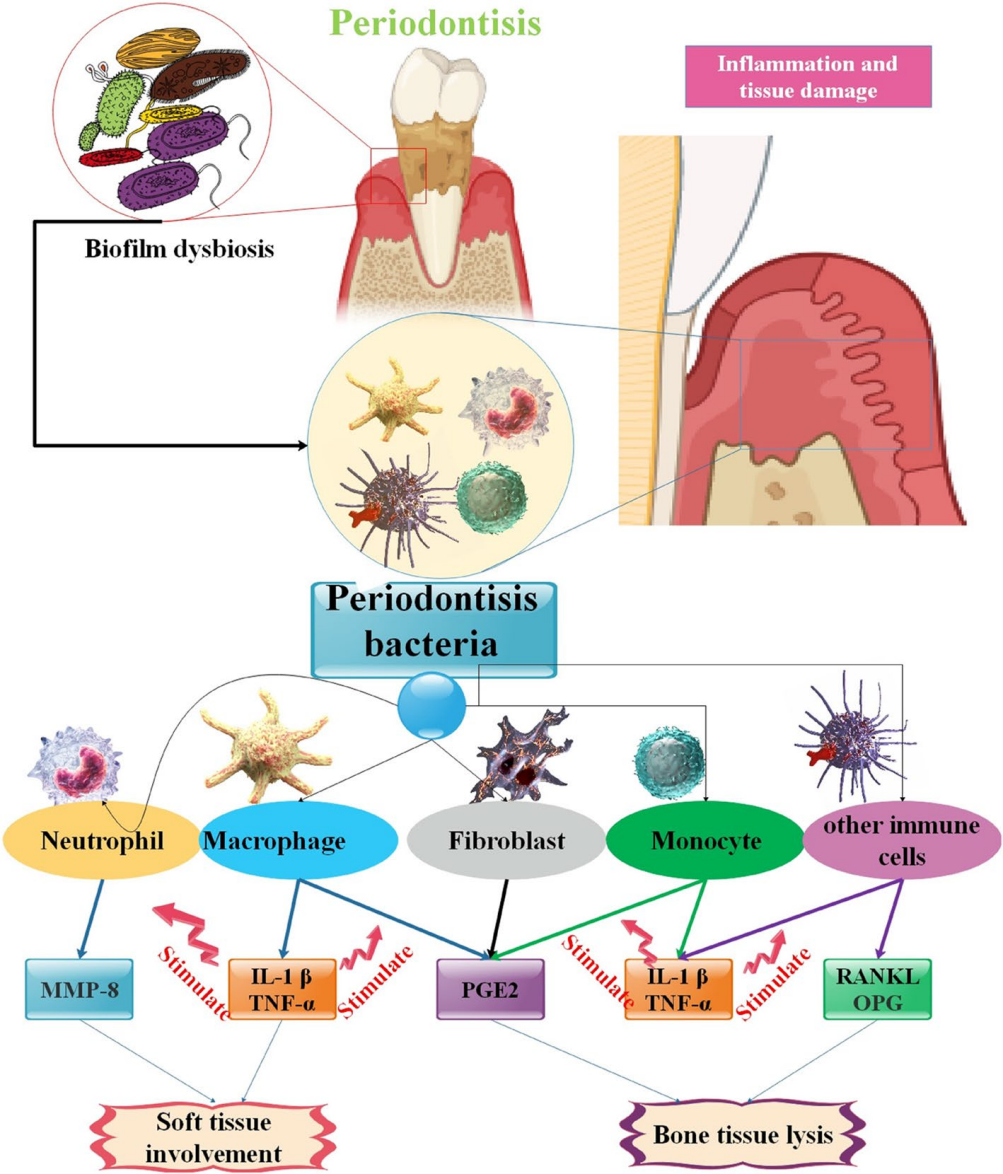
Periodontal disease biomarkers. Polymorphonuclear (PMN) leukocytes are the first defensive mechanism of periodontal tissues. Multiple products are released during the immunological response. MMP-8 is responsible for breaking type I, II, and III collagens. Several chemicals, including PGE2, IL-1, IL-6, and TNF-α, produced by Mø, fibroblasts, plasma cells, and T lymphocytes, have a role in activating osteoclasts. RANKL stimulates the differentiation of osteoclasts and prevents the programmed cell death of osteoclasts. In normal bodily settings, the RANKL generated by osteoblasts attaches to RANK receptors located on the surface of osteoclast precursors. RANKL is increased by Parathyroid hormone (PTH) and IL-1. Osteoprotegerin (OPG) is synthesized by fibroblasts and serves as a decoy receptor for RANK, hence suppressing osteoclastic activation. Abbreviations: IL-1 β represents interleukin -1, TNF-α stands for tumor necrosis factor α, PGE2 denotes prostaglandin E2, RANKL refers to receptor activator of nuclear factor kappa-B ligand, and OPG represents OPG [104, 105]

The constraints linked to salivary biomarkers include the variability in saliva flow rate across people, which is influenced by factors such as age, sex, medical condition, and circadian rhythm. The considerations unequivocally raise doubts about the precision and consistency of employing salivary biomarkers for detecting the first phases of periodontitis. The biomarker test should be conducted in real-time, enabling the instant monitoring of the patient's periodontal health inside the dentist's office. Furthermore, biomarkers should not only aid in the early detection of PDs but also facilitate predicting future risk using accessible and cost-effective methods [103]. Therefore, it can be said that although a few products have the potential to be beneficial and indicate which tissue components are in danger, the majority of test kits and biomarkers are expensive and provide little to no extra information. It's also evident that no single marker has been able to meet all the requirements needed to evaluate the clinical condition of the periodontium, and future studies may perhaps focus on creating "marker packages" while there are now attempts underway to design an ideal test, its practical use as a chairside diagnosis remains elusive. Thus, the main objective of periodontal research is to create a broad range of markers [102]. Therefore, more research is required to elucidate the true potential of a single biomarker or a combination of biomarkers in the diagnosis, development, and assessment of periodontitis [94] (Fig. 4).

## The use of nano-scale biosensors to detect periodontal disorders

Periodontal problems may be diagnosed using nano-scale biosensors. These sensors can detect and analyze chemicals in physiological fluids, including saliva, blood, and GCF [106]. An array of sodium-specific membranes, including magnetic nano-inclusions, using p-tertbutyl calix arene as an ionophore and polyvinyl chloride (PVC) as a polymeric matrix, has been successfully created. Consequently, sodium-specific sensors have been produced for the first time. A linear range was seen from 3.1 × 10^−5^ to 10^−1^ mol dm^−3^ for a sodium selective sensor based on PVC. The sensor exhibited a near Nernstian electrochemical response of 55.73 mV/decade with a response time of 45 s. Given their compact size, sensors have the potential to measure ions from the GCF inside the periodontal pocket directly. This eliminates the challenges associated with collecting a sufficient volume of fluid for analysis. This novel technology can detect changes in the levels of inorganic ions, which may aid in the early diagnosis and prevention of PD [107].

**Fig. 5:**
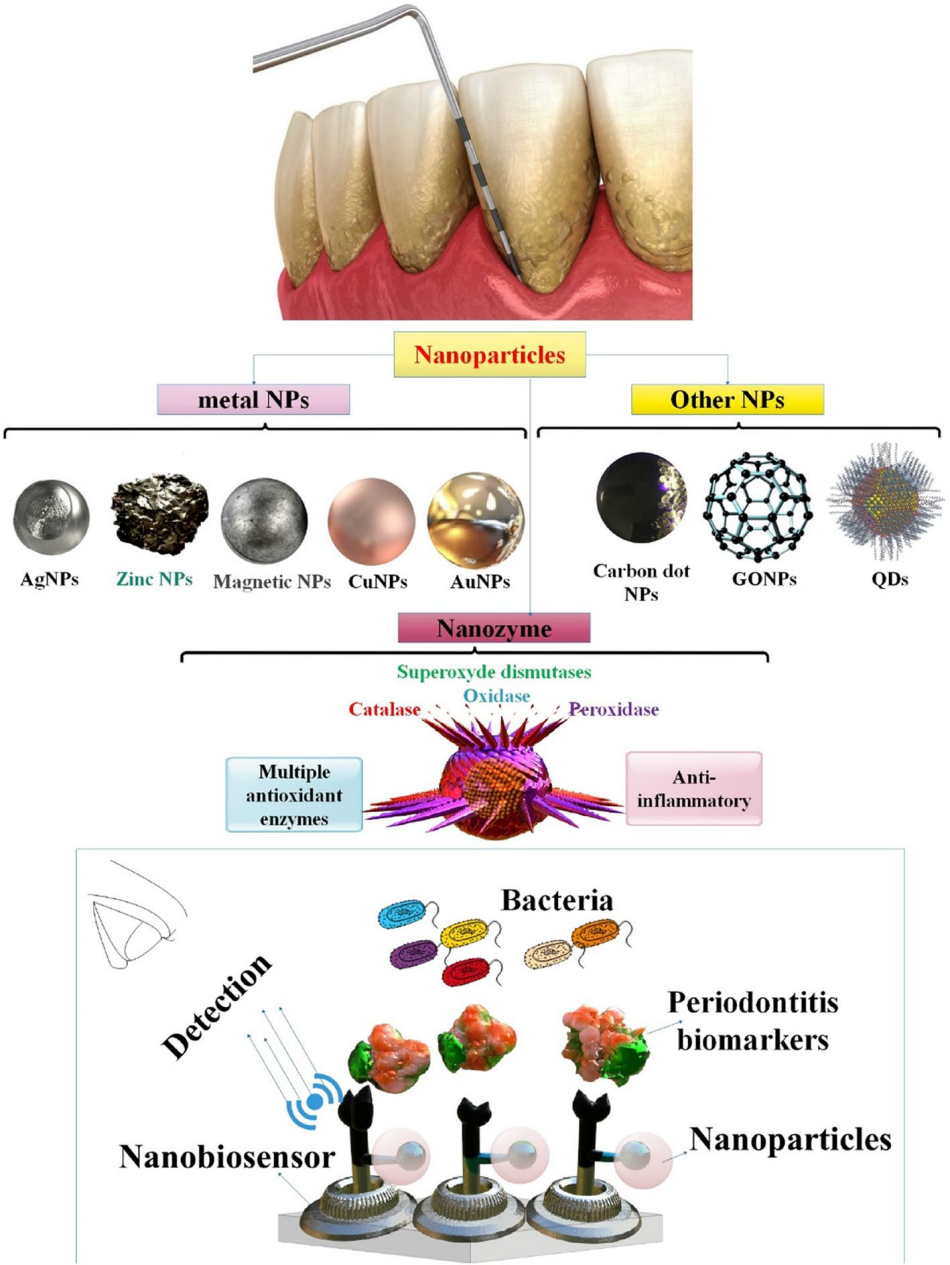
Employing various nanoparticles (NPs), including metal NPs, carbon-based NPs, quantum dots, and nanozymes, to detect periodontitis

Numerous varieties of NPs have been the subject of extensive research to date and have demonstrated considerable potential as nanodiagnostics for infectious diseases. The utilization of fluorescent NPs (e.g., QDs), magnetic NPs, and metallic NPs (e.g., Au and AgNPs) has proven to be effective in the imaging, tracking, and detection of a wide range of infectious microorganisms [108] (Fig. 5).

### Metal nanoparticles-based biosensors and their applications in periodontitis detection

Salivary indicators such as ALP and IL-1β play an essential role in this research for diagnosing PD, which causes damage to periodontal tissue and tooth loss. The geometrical modification of an Ag nanoplate transducer is the basis for the multicolorimetric ALP and IL-1β sensing platform the researchers developed in this study. To control the seed's shape transformation from triangular to hexagonal, rounded pentagonal, and spherical, the enzymatic activity of ALP is used to dephosphorylate p-aminophenol phosphate (p-APP) to p-aminophenol (p-AP). As a result, the localized surface plasmon resonance properties of the Ag nanoplate change from blue to yellow in response to ALP. The multicolor sensor has the lowest range of LOD for a state-of-the-art ALP sensor to date, with an ALP detection range of 0–25 U/L and a LOD of 0.0011 U/L. The sensor also shows a linear detection range of 0–250 pg/mL and a LOD of 0.066 pg/mL, two orders of magnitude lower than the monochromic conventional ELISA (LOD of 3.8 pg/mL) [109].

In a different investigation, it was shown that the primary culprit, *P. gingivalis*, is responsible for developing the illness by secreting gingipains, which are very harmful proteases. Gingipain activity detection in gingival fluid has the potential to pave the way for early diagnosis and therapy. A nanoplasmonic biosensor based on NPs that can detect gingipains' proteolytic activity is described here. After being treated with casein or IgG, AuNPs were allowed to self-assemble as a submonolayer in multiwell plates. It was using casein as a substrate causes proteases in the buffer to undergo proteolytic degradation, which blueshifts the localized surface plasmon resonance (LSPR) band by 1–2 nm in response to concentration and duration. The breakdown of the protein coating in bacterial supernatants caused proteins in the complex sample matrix to attach to the NPs unintendedly, shifting the LSPR band by around 2 nm. Only samples exhibiting gingipain action showed a significant LSPR change. The sensor's LOD of less than 0.1 μg/mL (4.3 nM) is much lower than the gingipain concentrations (about 50 μg/mL) seen in patients with severe chronic periodontitis. This study demonstrates the potential for creating affordable biosensors based on NPs that can quickly detect protease activity for chair-side periodontal diagnostics [110].

The study's researchers aimed to address the need for a sensing material that can accurately and selectively detect ppb-levels of methyl mercaptan (CH_3_SH) in exhaled breath, as its concentration increases slightly with the progression of PD. This material is crucial for the early diagnosis of periodontitis, as it allows for the discrimination of CH_3_SH from other volatile sulfur compounds (VSCs). The main objective is to suggest self-perceived PD sensors by strategically layering 30 nm-thick ZnO nanofilms with 3 nm-thick AuNPs using a two-step procedure, including atomic layer deposition and thermal evaporation techniques. The gas sensing performance is significantly enhanced by optimizing the ZnO material by size and density management of AuNPs. This improvement results in a gas response of 4.99% for 50 ppb of CH_3_SH and a detection limit as low as 50 ppb. The study confirmed the dependable and repeatable gas sensing performance of the Au NP-incorporated ZnO hybrid sensors for detecting ppb-level CH_3_SH in the presence of an H2S environment [111].

Detecting *P. gingivalis* using surface-enhanced Raman spectroscopy (SERS) is particularly difficult, especially when dealing with clinical samples. Activated AgNPs can be used as a SERS substrate to enhance the signals of *P. gingivalis* in diagnosing periodontitis. The activated AgNPs were used as the substrate, with the incorporation of the reducing agent (sodium borohydride) on two occasions, and the aggregating agent (Na^+^) to create a concentrated region of high reactivity, known as the "hot spot". This hot spot, resembling a protective layer or "mask", effectively captures bacteria on the surface [112].

This study presents a quick and efficient method for diagnosing periodontal infections caused by *P. gingivalis*. The technique employs gingipains, a protease unique to *P. gingivalis*, as a biomarker for detection. Gingipain-specific peptide substrates were used to mark magnetic nanobeads, which were then fixed on an Au biosensing platform via Au-thiol linkage. Consequently, the Au layer undergoes a color transformation and becomes black. After the immobilized substrates were cleaved by gingipains, the magnetic-nanobeads-peptide fragments were drawn towards a magnet, causing the golden surface color to reappear. The test has a high level of sensitivity and specificity. The device can identify as little as 49 colony-forming units per milliliter (CFU·mL^−1^) of *P. gingivalis* during 30 s. The analysis of saliva samples from patients with periodontitis and healthy controls demonstrated the assay's potential. The assay's simplicity and speed make it a very efficient POC device [15].

**Fig. 6:**
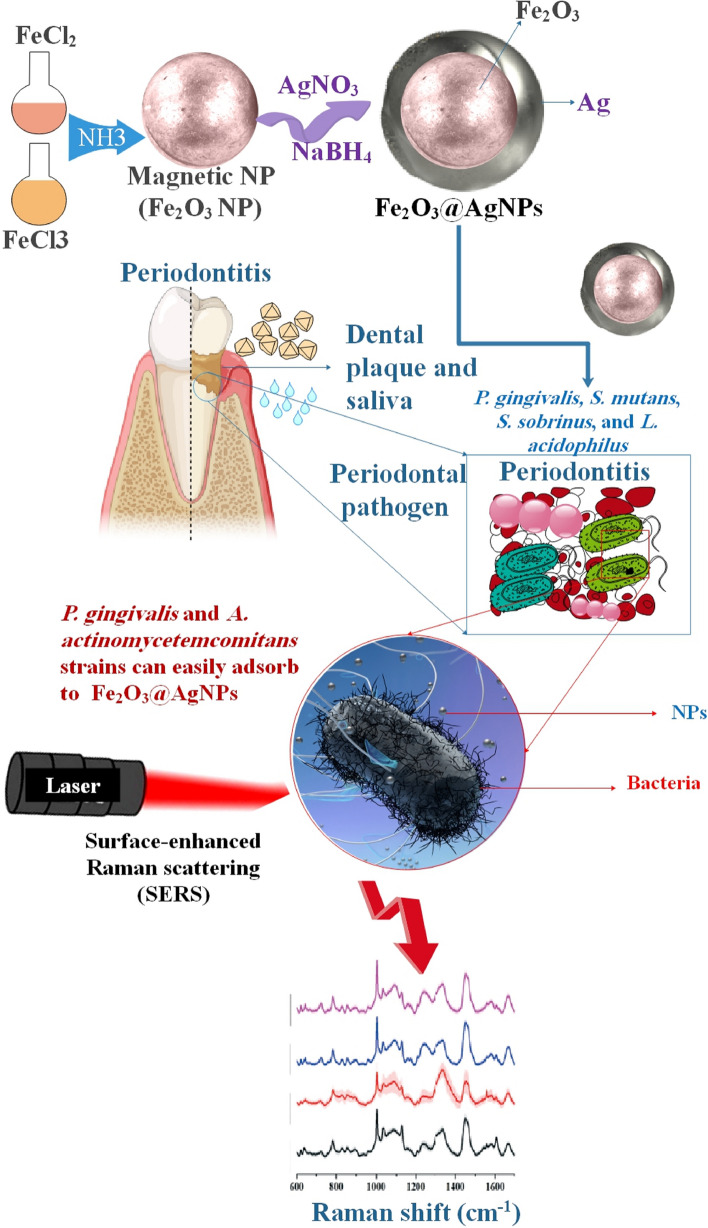
A magneto microfluidic sensor based on SERS for *A.actinomycetemcomitans* and *P. gingivalis* detection. The creation of a simple, quick, and accurate technique for identifying the bacteria causing periodontal disease was reported in this study. Furthermore, scientists demonstrated that strains of *A. actinomycetemcomitans* and *P. gingivalis* may be found and identified in clinical samples like human saliva. The Fe2O3@Ag magnetic NPs, the microfluidic chip, and the Si/Ag platform were used for this. Consequently, bacterial-NP aggregation formation, magnetic separation, and bacterial Raman signal augmentation were accomplished. Detecting low amounts of bacteria in clinical samples is more accessible by the comparatively high SERS signal acquired. Furthermore, researchers can distinguish 89% of the strains of *A. actinomycetemcomitans* from *P. gingivalis* using PCA on bacteria suspended in human saliva. Moreover, the sample may be created quickly and without labels [113]

*P. gingivalis*, a significant bacterium associated with periodontal disease (periodontitis), has been found in the brains of people with Alzheimer's disease. Furthermore, via SERS measures, *P. gingivalis* may be differentiated from *A. actinomycetemcomitans*, another prevalent periodontal pathogen, and from widespread oral *Streptococcus spp*. Moreover, scientists have shown that several strains of *P. gingivalis* and *A. actinomycetemcomitans* may readily adhere to Ag-coated magnetic NPs (Fe_2_O_3_@AgNPs). Hence, it is feasible to use magnetic forces to isolate the examined bacteria from other constituents of a sample using the microfluidic chip. To further amplify the Raman signal, the NPs adhered to bacterial cells were magnetically drawn towards the Si/Ag SERS platform. Subsequently, the SERS spectra could be documented. An efficient process like this may greatly aid in quick medical diagnosis, enabling the initiation of the proper pharmaceutical treatment to avoid the progression of periodontitis and related comorbidities, such as Alzheimer's disease (Fig. 6) [113].

The identification and timely diagnosis of oral disorders such as dental caries and periodontitis might be accomplished by monitoring the release of VSCs in the oral cavities. This study presents a novel method for precisely identifying dental lesion locations. It utilizes a fluorescent mouthguard made of a nanocomposite material called zinc oxide–poly(dimethylsiloxane) (ZnO-PDMS). The mouthguard can detect the specific release of VSCs in the affected area. The ZnO-PDMS mouthguard has exceptional sensitivity and specificity in detecting VSCs while also maintaining a stable fluorescence signal. It possesses favorable biocompatibility and little toxicity when exposed to normal physiological conditions. Subsequently, the ZnO-PDMS mouthguard, which may be worn, is shown to have the capability to detect the specific positions of injury sites in individuals accurately. By using image analysis, the mouthguards effectively identify the exact positions of dental caries, enabling straightforward detection of concealed dental lesion areas that are often overlooked by dentists. Due to cheap cost, long-term stability, and robust patient compliance, the suggested wearable mouthguard is appropriate for large-scale manufacture. It allows broadly applicable, preliminary but accurate screening of dental lesions before dental clinics and regular physical exams [114].

The precise identification of the MMP-8 enzyme would provide a dependable quantitative evaluation of periodontal "grading," a crucial indicator of PD progression. This study discusses the creation of a new kind of sensor that uses voltammetry to detect salivary MMP-8. The sensor is designed to be used at the POC, meaning it may be used in a clinical setting without laboratory equipment. The electrochemical platform used a GPH screen-printed electrode (SPE) modified with AuNSs and antibodies specific to the MMP-8 protein (anti-MMP-8). The voltammetric sensor demonstrated excellent performance, with a linear range spanning from 2.5 to 300 ng mL − 1. The LOD was determined to be 1.0 ± 0.1 ng mL^−1^, and the sensitivity was measured at 0.05 µA mL cm^−2^ ng^−1^. The biosensor, being disposable and economical, necessitates just a tiny amount of test media and a brief preparation period. Consequently, it is an exceedingly appealing biosensor for detecting MMP-8 in human saliva [115].

The concentration of H_2_O_2_ in saliva and GCF may serve as an indicator of the severity of periodontitis. A flexible electrochemical sensor for detecting H_2_O_2_ was created using a screen-printed electrode (SPE) that was enhanced with a Cu NP-anchored Cu-based metal–organic framework (Cu NPs@Cu-MOF) and Ti carbide nanosheets (Ti_3_C_2_T_x_ NSs). Significantly, this particular sensor, known as an SPE sensor, has advantageous flexibility that makes it suitable for precise attachment to the human body, regardless of its varying profile. In addition, the nanohybrid demonstrates exceptional electrocatalytic activity due to the large surface area of tiny Cu NPs, the hierarchical structure of Cu-metal organic framework (Cu-MOF), the enhanced rate of electron transfer from Ti carbide nanosheets (Ti_3_C_2_T_x_ NSs), and the remarkable synergistic impact of the combination of Cu NPs inside the Cu-MOF and Ti_3_C_2_T_x_ NSs. The sensor exhibits a high sensitivity of 254.9 μA mM^−1^ cm^−2^ (0.2–26.1 μM) and a low detection limit of 84.5 nM. About its viability, the created sensor is capable of detecting and monitoring the emission of H_2_O_2_ at the cellular level. Furthermore, it can differentiate between those who are in good health and those who have gingivitis and periodontitis by analyzing the levels of H_2_O_2_ in samples of saliva and GCF. Therefore, the sensing platform that has been built has excellent potential as an effective tool for early detection of periodontitis [116] (Table 1).

**Table 1:** Metal nanoparticles in periodontitis detection

Metal nanoparticle	Biomarker	Platform	LOD and other details	Effects	Ref
Cu NPs@Cu-MOF and Ti carbide nanosheets (Ti_3_C_2_T_x_ NSs)	H_2_O_2_ in saliva and GCF	Versatile electrochemical platform with screen printing	Sensitivities of 254.9 μA mM^−1^ cm^−2^ (0.2–26.1 μM) and a detection limit of 84.5 nM	The concentration of H_2_O_2_ in saliva and GCF may serve as an indicator of the severity of periodontitis. Furthermore, it can differentiate between those who are in good health and those who have gingivitis and periodontitis by analyzing the levels of H_2_O_2_ in samples of saliva and GCF	[116]
Ag Nanoplates	Alkaline phosphatase (ALP) and interleukin-1beta (IL-1β)	Multicolor sensor	ALP detection range of 0–25 U/L with a limit of detection (LOD) of 0.0011 U/L, IL-1β for multicolor signaling, and it exhibits a linear detection range of 0–250 pg/mL and a LOD of 0.066 pg/mL,	The geometrical modification of an Ag nanoplate transducer is the basis for the multicolorimetric ALP and IL-1β sensing platform the researchers developed in this study With a recovery of 100.9% in actual human saliva, the ALP multicolor sensor demonstrates excellent selectivity, demonstrating its dependability and appropriateness for easily accessible periodontal diagnostics with multivariate signal readout	[109]
AuNPs	*P. gingivalis*	Refractometric nanoplasmonic sensors	Less than 0.1 μg/mL (4.3 nM) well below gingipain concentrations detected in severe chronic periodontitis cases (∼50 μg/mL)	After being treated with casein or IgG, AuNPs were allowed to self-assemble as a submonolayer in multiwell plates. The breakdown of the protein coating in bacterial supernatants caused proteins in the complex sample matrix to attach to the NPs unintendedly, shifting the LSPR band by around 2 nm. Only samples exhibiting gingipain action showed a significant LSPR change	[110]
ZnO NPs with AuNPs	methyl mercaptan (CH_3_SH) among volatile sulfur compounds (VSCs)	Chemiresistive gas sensors	the significant enhancement of the gas sensing performance with 4.99% gas response for 50 ppb of CH_3_SH and a detection limit down to 50 ppb	The main objective is to suggest self-perceived PD sensors by strategically layering 30 nm-thick ZnO nanofilms with 3 nm-thick AuNPs using a two-step procedure, including atomic layer deposition and thermal evaporation techniques	[111]
Ag NPs	*P. gingivalis*	Surface-enhanced Raman spectroscopy (SERS)	LOD = 10^5^ CFU/mL, the platform can quickly identify common oral bacteria by combining with machine learning	The study included integrating advanced mechanical learning techniques with accurately categorizing four distinct oral bacteria, namely *P. gingivalis*, *E. faecalis*, *S. aureus*, and *S. mutans*. The unlabeled, user-friendly, and environmentally friendly SERS detection technique for oral bacteria has significant promise for clinical early diagnosis and prediction of disease progression in PD	[112]
Magnetic-nanobeads	Gingipains	Colorimetric assay and effective point-of-care device	It can detect as little as 49 CFU·mL^−1^ of *P. gingivalis* within 30 s	The technique employs gingipains, of proteases unique to *P. gingivalis*, as a biomarker for detection. After the immobilized substrates were cleaved by gingipains, the magnetic-nanobeads-peptide fragments were drawn towards a magnet, causing the golden surface color to reappear. Gingipain-specific peptide substrates were used to mark magnetic nanobeads, which were then fixed on a gold biosensing platform via gold-thiol linkage	[15]
Fe_2_O_3_@AgNPs	*P. gingivalis* and *A. actinomycetemcomitan*	surface-enhanced Raman scattering (SERS)	LOD = 10^3^ cfu/mL. Additionally, the PCA performed for bacteria suspended in human saliva allowed us to separate *P. gingivalis* from *A. actinomycetemcomitans* strains with 89% accuracy	Scientists have shown that several strains of *P. gingivalis* and *A. actinomycetemcomitans* may readily adhere to Ag-coated magnetic NPs (Fe_2_O_3_@AgNPs). Multidisciplinary research shows that *P. gingivalis* may be readily detected using SERS	[113]
ZnO-PDMS	Volatile sulfur compounds (VSCs)	A transparent, wearable fluorescent mouthguard for high-sensitive visualization	The ZnO-PDMS mouthguards worn by volunteers for 7 h: fluorescence images	The ZnO-PDMS mouthguard has exceptional sensitivity and specificity in detecting VSCs while also maintaining a stable fluorescence signal. It possesses favorable biocompatibility and little toxicity when exposed to normal physiological conditions. Subsequently, the ZnO-PDMS mouthguard, which may be worn, is shown to have the capability to detect the specific positions of injury sites in individuals accurately	[114]

### Carbon nanoparticles-based biosensors and their applications in periodontitis detection

Renewable and environmentally friendly carbon-based nanomaterials, such as GPH, GPH oxide, reduced graphene oxide (GO), GPH QDs, carbon nanotubes, MXenes, and carbides, possess exceptional physical, chemical, and biological characteristics. CBMs, known for their substantial surface area and robustness, have significantly impacted the fields of dentistry and biomedicine [117]. Carbon nanomaterial-based ECL signaling probes can identify important biomarkers in artificially contaminated human blood samples. This suggests that they might be used for diagnosing infectious illnesses and malignancies [118].

Recent developments in the dental and oral research applications of GO and functionalized GO. A multitude of exceptional physical, chemical, optical, electrical, and mechanical characteristics are exhibited by GO [119]. Oxygenated functional groups and a broad surface area provide GO with remarkable metal-ion and organic-species interaction capabilities [120]. Chlorogenic acid (CGA), a phenolic acid found in coffee, has been recognized as a potent compound in combating oxidative stress and inflammation, according to research. Additionally, its ability to disrupt PD and facilitate healing makes it a very intriguing target for therapeutic development. Nevertheless, the current techniques for CGA detection have limited practical use in the purification and subsequent pharmacological investigation in stomatology, mainly owing to their inadequate precision and efficiency. Hence, it is essential to identify a robust methodology to assess CGA for a comprehensive anti-PD investigation accurately. The researchers described a simple and adjustable method for creating Pt@Pd nanowires (NWs) with a core–shell structure that is not tightly packed. These nanowires exhibit excellent electrocatalytic activity. Furthermore, using polyethyleneimine (PEI)-capped reduced graphene oxide (rGO) nanoflakes facilitated the formation of a network structure consisting of interweaved Pt@Pd nanowires. This structure effectively shielded hemin from self-destruction and resulted in Pt@Pd NWs-Hemin-PEI-rGO nanohybrids possessing a substantial electroactive surface area and exceptional electrochemical properties for detecting CGA. The electrochemical sensor, which does not need enzymes, is constructed using Pt@Pd nanowires functionalized with Hemin, PEI, and rGO. This sensor has excellent sensitivity for detecting trace amounts of CGA, with a detection limit of 7.8 nM. Furthermore, it has a broad linear detection range from 0.5 μM to 4 mM. The sensor's remarkable sensitivity and selectivity allowed for accurate measurements of CGA in soft drinks and coffee, with consistent findings comparable to those obtained using HPLC. The sensor's commendable performance enable it to be used for quality control and investigation of medication metabolism in PD therapies [121].

### Quantum dots-based biosensors and their applications in periodontitis detection

Due to their unique and superior electronic and optical properties, such as high brightness, excellent stability under light exposure, narrow and adjustable emission spectrum, broad absorption spectrum, versatile surface modification, a significant difference between excitation and emission wavelengths, and distinctive ability to convert light into electrical energy, semiconductor QDs with sizes ranging from 2 to 10 nm have been regarded as promising and attractive components for the development of efficient microRNA detection assays. These assays offer high sensitivity, excellent selectivity, rapid detection, and simplicity [25]. QDs are nanocrystals made of semiconducting materials with great potential for tagging and detecting germs. Researchers aimed to use QD-based primary immunofluorescence to label bacterial cells in both in vitro and in vivo biofilms. The use of QD-based immunofluorescence in studying biofilms in laboratory settings and living organisms will contribute to the understanding of bacterial community structure and enable research into the relationships among different bacterial species within these communities [122].

It would be ideal to develop a sensitive POC platform for identifying functional dyskinesia, periodontitis, and hidden caries. One possible method for non-invasive clinical diagnosis is breath analysis. In conjunction with tracking movement abnormalities in the craniofacial region, the accuracy of diagnosing illnesses may be more significant increased. In a study, researchers a multifunctional heterostructure called the WS_2_/MoS_2_ heterostructure—which combines n-type MoS_2_ QDs with p-type WS_2_ nanosheets—is built to enable multi-parameter sensing using a variety of substrates. At room temperature (25 °C) with UV assistance, the gas sensor based on an enhanced WS_2_/MoS_2_ heterostructure (W: Mo = 300:1) has a greater response (73.1% to 1 ppm) and a lower LOD of 10 ppb. This sensor has a broad sensing range, capable of detecting strains ranging from 10 to 160% and pressures ranging from 5 to 250 kPa. Furthermore, it is proficient in detecting minor and significant oral and maxillofacial motions. Ultimately, a versatile wearable platform is effectively utilized to distinguish the respiratory biomarkers of healthy persons from simulated exhaled breath in patients with caries and periodontitis, as well as to differentiate the bite force of incisors and molars in various populations [123].

Investigators described a peptide-QDs composite fluorescent probe that enables early detection of susceptible sites conceals early carious lesions of dental caries and provides high-performance imaging of *S. mutans*. The biocompatibility, minimal biotoxicity, and high fluorescence stability of this fluorescent probe in physiological environments are all noteworthy attributes. By fluorescently labeling *S. mutans*, which is accountable for the onset of dental caries, it is capable of explicitly identifying and localizing dental lesion sites. The integration of visible fluorescence analysis into the screening process facilitates the detection of concealed dental lesions that might go unnoticed. For accurate screening of dental disorders, the peptide-QDs composite fluorescent probe can be extensively utilized in routine dental examinations due to its low synthesis cost, stable storage, and straightforward imaging method [124].

GQDs have gained significant prominence as a novel category of fluorescent carbon materials due to their promising applications and outstanding characteristics [125]. GQDs are distinguished by many characteristic qualities. GQDs have demonstrated promise in numerous applications, including sensing, energy devices, drug delivery, bioimaging, photothermal, and photodynamic therapy, among others [126].

The estimation of lactoferrin is gaining prominence as an emerging biomarker in the diagnosis of PD, according to one study. Consequently, a GQDs@MnO_2_-NS nanoprobe was developed, which utilized manganese dioxide nanosheets (MnO_2_-NS) and straightforward, exceptionally sensitive, and selective fluorescent turn 'Off–On' mechanisms. The addition of MnO2-NS in this study effectively suppressed the fluorescence of GQDs via inner filter effects (IFE) and a robust interaction between the GQDs and MnO_2_-NS. The presence of lactoferrin resulted in the destruction of MnO2-NS and the resurgence of fluorescence emission from GQDs. This may have been caused by a redox reaction between lactoferrin and the prepared MnO2-NS. In this context, the nanoprobe provides a broad range of concentrations and a low LOD of 1.69 ng/mL and 5 to 1600 ng/mL, respectively. As fabricated, the GQDs@MnO_2_-NS nanoprobe sensor exhibited excellent stability, reproducibility, and selectivity concerning lactoferrin, thereby confirming the biosensor's applicability. Consequently, the GQDs@MnO_2_-NS nanoprobe will provide a straightforward sensor with sufficient sensitivity to detect lactoferrin in a highly responsive and selective manner [127].

Scientists are researching the potential use of Nano-GO QDs (GOQDs) as fluorescent markers for human periodontal ligament stem cells (hPDLSCs). The project seeks to assess the safety and effectiveness of GOQDs as live cell fluorescence markers. The hPDLSCs were cultured with several doses of GOODs (0, 10, 25, and 50 μg/mL) for 24 and 72 h. The proportional distribution of the G1, G2, and S phases did not differ significantly between the two GOQD concentrations (0 g/mL and 50 g/mL). Fluorescent images demonstrated that at a concentration of 50 μg/mL, GOQDs were capable of penetrating the cell membrane and augmenting the fluorescence intensity. It was verified that GOQDs possessed favorable biocompatibility and could be employed to label living cells of hPDLSCs [128].

### Nanozymes-based biosensors and their applications in periodontitis detection

The field of dentistry is progressing in parallel with the advancements in materials science. Nanozymes are increasingly being studied and used in nanocatalytic medicine, particularly in oral research and application. To emphasize the significant role of nanozymes in promoting dental health, researchers conduct a comprehensive analysis of the overall advancements in multi-functional nanozymes for various oral diseases. These diseases encompass the treatment of dental caries, dental pulp diseases, oral ulcers, and peri-implantitis. Additionally, nanozymes are utilized for monitoring oral cancer, oral bacteria, and ions, as well as facilitating the regeneration of both soft and hard oral tissues [129, 130].

**Fig. 7:**
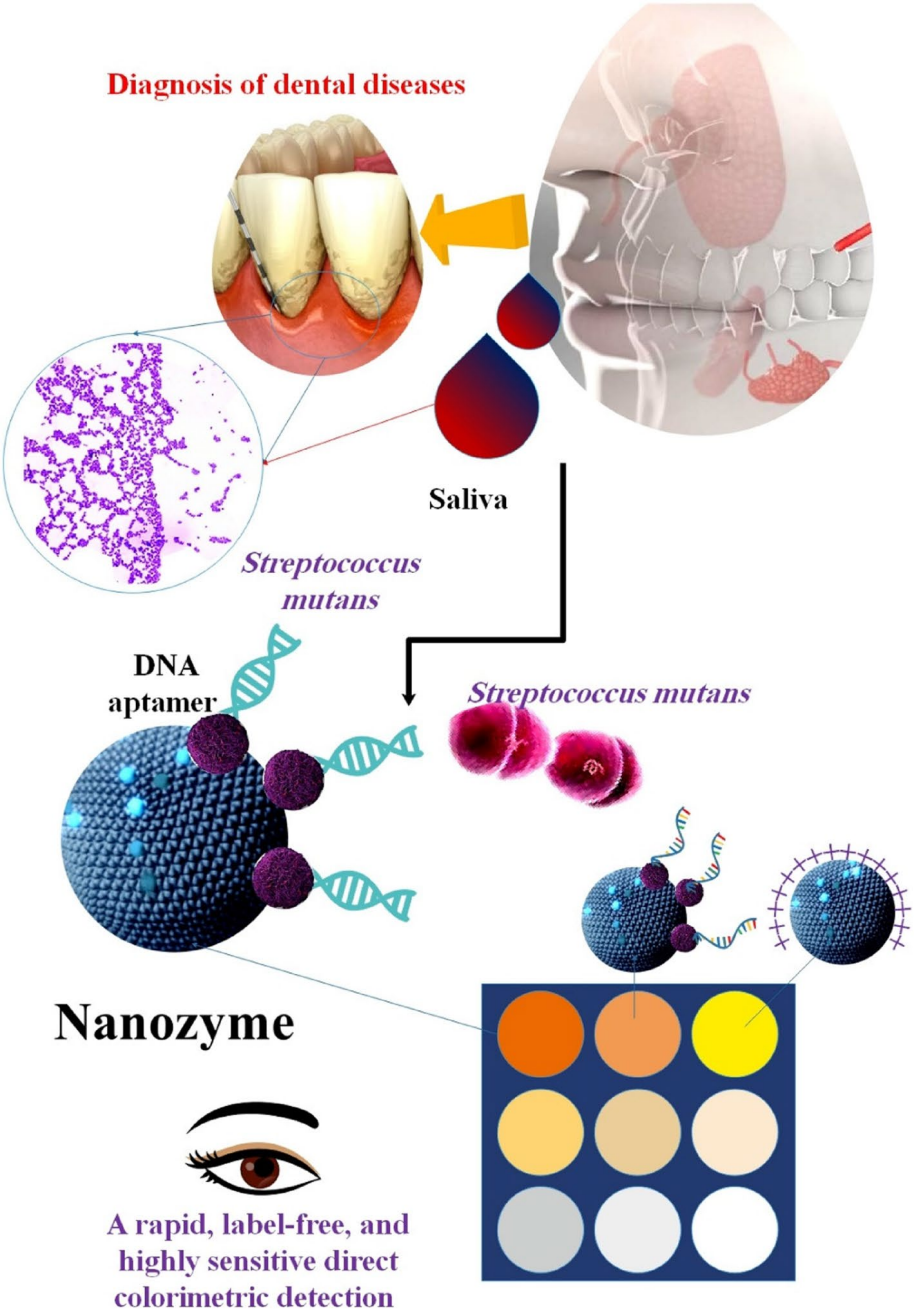
Three biosensors (DNA-engineered nanozymes) are used to detect *S. mutans* in saliva samples. Out of the three nanosystems, the one constructed with affinity-coupling and DNAzyme included showed the best specificity and the greatest sensitivity. A nanosystem like that has several built-in advantages. The DNA aptamer's role as a molecular recognition element and target sticky material results in the suppression of bacterial enzymatic activity. The identified process was then completed in 15 min, saving hours of molecular extraction or days of traditional culturing time. It was an easy and quick process. The colorimetric signal response of all three biosensors showed a concentration-dependent pattern, wherein the absorbance gradually decreased as the *S. mutans* concentration rose [131]

Researchers conducted a separate investigation by creating a DNA-engineered nanozyme interface to quickly and accurately identify tooth germs. Scientists used DNA aptamer as molecular recognition tools and sticky surfaces to modify the nanozyme. Various immobilization techniques and DNA designs were used to create DNA nanoscale biointerfaces, which were used to control the enzymatic and biological characteristics of the nanozyme systems. The functional biointerfaces enhanced the bacteria's ability to reach the nanozyme surface, resulting in a wide variety of signal changes when the DNA probe density is optimized. The nanozymes modified with DNA exhibit a fast, without labeling, and susceptible method for directly detecting *S. mutans* via color changes. The detection limit is 12 CFU mL^–1^, and it can effectively distinguish *S. mutans* from other bacteria in the mouth. The researchers have successfully shown the use of this biological nanointerface in detecting dental bacteria in samples of saliva, highlighting its potential in the clinical realm for preventing and diagnosing dental illnesses [131] (Fig. 7) (Table 2).

**Table 2:** Several non-metallic nanoparticles in periodontitis detection

Type of Nanoparticles	Biomarker	Platform	LOD and other details	Time monitoring	Explain	Ref
DNA-engineered nanozyme	*S. mutans*	colorimetric	12 CFU mL^–1^	15 min	The functional bio-interfaces enhanced the bacteria's ability to reach the nanozyme surface, resulting in a wide variety of signal changes when the DNA probe density is optimized. The nanozymes modified with DNA exhibit a fast, without labeling, and susceptible method for directly detecting *S. mutans* via color changes	[131]
Pt@Pd nanowires (NWs)	Chlorogenic acid (CGA)	Electrochemical sensor	LOD 7.8 nM and a wide linear range of 0.5 μM to 4 mM	Trigger electrochemical reactions faster	This structure effectively shielded hemin from self-destruction and resulted in Pt@Pd NWs-Hemin-PEI-rGO nanohybrids possessing a substantial electroactive surface area and exceptional electrochemical properties for detecting CGA. The electrochemical sensor, which does not need enzymes, is constructed using Pt@Pd nanowires functionalized with Hemin, PEI, and rGO	[121]
Graphene (GPH) screen-printed electrode (SPE) functionalized by AuNSs	Salivary MMP-8	Voltammetric immunosensor	Alinear range of 2.5–300 ng mL^−1^, a LOD value of 1.0 ± 0.1 ng mL^−1^ and a sensitivity of 0.05 µA mL cm^−2^ ng^−1^	Fast and sensitive detection	This text discusses the creation of a new kind of sensor that uses voltammetry to detect salivary MMP-8. The electrochemical platform used a graphene screen-printed electrode (SPE) modified with AuNSs and antibodies specific to the MMP-8 protein (anti-MMP-8)	[115]
n-type MoS_2_ quantum dots	Distinguishes the respiratory biomarkers and bite force	Point-of-care platform	73.1% to 1 ppm NO_2_ with 10 ppb LOD under UV. Sensing ranges are 10%-160% strain and 5–250 kPa pressure	Fast detection	Researchers a multifunctional heterostructure called the WS_2_/MoS_2_ heterostructure—which combines n-type MoS_2_ QDs with p-type WS_2_ nanosheets—is built to enable multi-parameter sensing using a variety of substrates	[123]
Peptide-QDs composite	*S. mutans*	Peptide-QDs composite fluorescent probe	-	Fast detection	Peptide-QDs composite fluorescent probe that enables early detection of susceptible sites conceals early carious lesions of dental caries and provides high-performance imaging of *S. mutans*	[124]
GQDs@MnO_2_-NS nanoprobe	Lactoferrin	Fluorescence turns “On–Off-On” nanoprobe	1.69 ng/mL and 5 to 1600 ng/mL	Fast detection	A GQDs@MnO_2_-NS nanoprobe was developed, which utilized manganese dioxide nanosheets (MnO_2_-NS) and straightforward, exceptionally sensitive, and selective fluorescent turn 'Off–On' mechanisms	[127]
Nano-graphene oxide QDs (GOQDs)	hPDLSCs	Fluorescent imaging	50 μg/mL	hPDLSCs were incubated with different concentrations of GOODs (0, 10, 25, and 50 μg/mL) for 24 h and 72 h	The project seeks to assess the safety and effectiveness of GOQDs as live cell fluorescence markers. Fluorescent images demonstrated that at a concentration of 50 μg/mL, GOQDs were capable of penetrating the cell membrane and augmenting the fluorescence intensity	[128]

## Future and perspective nanobiosensors in the diagnosis of periodontitis

Regrettably, its significance in diagnosing oral maladies, including periodontitis, remains undervalued. Regarding managing biomarkers, researchers believe it is crucial to emphasize that dentistry is presently out of step with contemporary practices. A cultural deficiency exists in the current state of novel diagnostic strategy development, necessitating a global strategy that promotes a contemporary cultural approach to oral disease diagnosis [104]. While the utilization of NPs as a method for detecting periodontitis is encouraging, certain limitations must be considered. Validation is required for candidate biomarkers identified via pilot studies that exhibit differential expression. Due to the lack of research in this area, a meaningful sample size calculation was not possible. An area of concern that requires attention in these studies is the age disparity among the various groups; therefore, to eliminate age as a potential confounding variable, it must be corrected. Furthermore, while the development of biomarkers remains of utmost importance, it is equally imperative to devise appropriate treatment and prevention strategies for patients who exhibit positive results for said biomarkers [100, 132, 133].

The field of nanotechnology has made significant strides in the advancement of biosensors. A diverse range of detection techniques have been developed utilizing biosensors, such as electrochemical biosensors, optical biosensors, photonic crystal biosensors, colorimetric biosensors, SPR biosensors, and fluorometric biosensors. The public is becoming increasingly interested in biosensors due to their numerous advantages, which include high sensitivity, selectivity, repeatability, low cost, minimal sample requirement, and a compact device that is easy to handle and operate. Certain nanoprobes that have been intricately designed have demonstrated detection limits as low as femtomolar, signifying an increase in sensitivity over conventional detection techniques. Notwithstanding the favorable performance and triumphant demonstration of nanobiosensor-based periodontitis detection, numerous obstacles present substantial impediments to the clinical implementation of these diagnostic techniques. One crucial aspect to consider is the meticulous and accurate manipulation of NP size, as the properties exhibited by NPs are significantly influenced by their dimensions. When developing optical nano biosensors, numerous factors must be considered, including but not limited to minimal background fluorescence, low photodamage to virus oligonucleotide hybridization with the probe, high photostability, and low phototoxicity of the probes. Furthermore, it is imperative to consider the concerns about the toxicity, reproducibility, and throughput of the biosensors. Nanobiosensors have generated considerable interest due to their capacity to identify a wide variety of analytes at exceedingly minute concentrations. New forms of POC, ultrasensitive, low-cost, robust, and dependable diagnostic techniques are likely to emerge as this field advances. Thus, the future holds great promise for an optical biosensing system based on nanomaterials that possesses exceptional stability, biocompatibility, reproducibility, and high sensitivity [134].

## Conclusion

Periodontitis is a prevalent global illness that requires efficient diagnoses, prompt treatment, and control of its development to avoid tooth loss. There has been a longstanding interest in developing a diagnostic tool for PD that can identify its onset and development, evaluate the effectiveness of treatment, and measure the vulnerability to future disease progression. The potential of enzymes, proteins, and Ig, which are significant components of saliva, as biomarkers for PDs has been well recognized and intensively studied. The advancement of nanotechnology has led to potential uses in several fields, including developing biosensors. Electrochemical biosensors based on nanomaterials have been a central focus in diagnostics. The investigation of nanomaterials as possible remedies has been spurred by the growing requirement for increased sensitivity and decreased detection limits. The advancement of diverse biosensors, including affinity-based nano-biosensors, GPH affinity-based biosensors, optical nano-biosensors, SPR-based optical nano-biosensors, and electrochemical nano-biosensors, has facilitated the rapid and sensitive identification of periodontitis. A variety of NPs, including Au and Ag, carbon-based NPs, QD, and ZnO, have been extensively studied because of their distinct features that are crucial for their application in biosensors for the early detection of periodontitis. This is primarily due to their exceptional electrical conductivity, high SA: V ratio, and remarkable chemical stability. Nevertheless, the vast majority of research on dental nanomaterials has been conducted in vitro, with little attention paid to or demonstrated f the (positive) impact of the nanomaterials in vivo. The reported material's nano-improvement effect may be negligible or contentious in certain instances. Thus, further investigation into the use of these novel NPs to detect periodontitis will aid in creating a specialized method for early periodontitis diagnosis that is inexpensive, precise, and rapid.

## Data Availability

No datasets were generated or analysed during the current study.
